# Chitosan-Based Films with 2-Aminothiophene Derivative: Formulation, Characterization and Potential Antifungal Activity

**DOI:** 10.3390/md20020103

**Published:** 2022-01-26

**Authors:** Verônica da Silva Oliveira, Meriângela Miranda da Cruz, Gabriela Suassuna Bezerra, Natan Emanuell Sobral e Silva, Fernando Henrique Andrade Nogueira, Guilherme Maranhão Chaves, José Lamartine Soares Sobrinho, Francisco Jaime Bezerra Mendonça-Junior, Bolívar Ponciano Goulart de Lima Damasceno, Attilio Converti, Ádley Antonini Neves de Lima

**Affiliations:** 1 Department of Pharmacy, Federal University of Rio Grande do Norte, Natal 59012-570, RN, Brazil; veronicasoliver47@gmail.com (V.d.S.O.); meriangela.c@gmail.com (M.M.d.C.); gabisuassuna.08@gmail.com (G.S.B.); natan.farmacia@gmail.com (N.E.S.e.S.); fernando.nogueira@nuplam.ufrn.br (F.H.A.N.); guilherme.chaves@ufrnet.br (G.M.C.); 2 Department of Pharmaceutical Sciences, Federal University of Pernambuco, Recife 50740-520, PE, Brazil; joselamartine@hotmail.com; 3 Laboratory of Synthesis and Drug Delivery, State University of Paraíba, João Pessoa 58071-160, PB, Brazil; franciscojbmendonca@yahoo.com.br; 4 Department of Pharmacy, Center for Biological and Health Sciences, State University of Paraíba, Campina Grande 58429-600, PB, Brazil; bolivarpgld@hotmail.com; 5 Department of Civil, Chemical and Environment Engineering, Pole of Chemical Engineering, University of Genoa, I-16145 Genoa, Italy

**Keywords:** films, chitosan, 2-aminothiophene derivative, antifungal activity

## Abstract

In this study, films of chitosan and 2-amino-4,5,6,7-tetrahydrobenzo[*b*]thiophene-3-carbonitrile (6CN), a 2-aminothiophene derivative with great pharmacological potential, were prepared as a system for a topical formulation. 6CN-chitosan films were characterized by physicochemical analyses, such as Fourier-transform infrared spectroscopy (FTIR), differential scanning calorimetry (DSC), thermogravimetric analysis (TGA), X-ray diffraction (XRD), and scanning electronic microscopy (SEM). Additionally, the antifungal potential of the films was evaluated in vitro against three species of *Candida* (*C. albicans*, *C. tropicalis*, and *C. parapsilosis*). The results of the FTIR and thermal analysis showed the incorporation of 6CN in the polymer matrix. In the diffractogram, the 6CN-chitosan films exhibited diffraction halos that were characteristic of amorphous structures, while the micrographs showed that 6CN particles were dispersed in the chitosan matrix, exhibiting pores and cracks on the film surface. In addition, the results of antifungal investigation demonstrated that 6CN-chitosan films were effective against *Candida* species showing potential for application as a new antifungal drug.

## 1. Introduction

The increase in fungal infections poses a serious threat to human health and life [1], especially in immunocompromised individuals with underlying diseases such as tuberculosis, leukemia, acquired immunodeficiency syndrome (AIDS), cancer, or diabetes, and patients undergoing chemotherapy or transplantation [2,3,4]. The pathogens *Aspergillus fumigatus*, *Candida albicans*, and *Cryptococcus neoformans* are associated with higher mortality rates in the immunocompromised population [1,2].

The high incidence of fungal infections is a clinical challenge due to difficulties in diagnosis and treatment [5]. Many available drugs have low efficacy, high toxicity, disadvantages in relation to their spectrum of activity, low potency (usually fungistatic), and inadequate pharmacokinetic properties [3,4]. In addition, conventional antifungals have become ineffective due to the emergence of drug-resistant strains, thereby making treatment more complex and reducing the number of drugs on the market [2,6]. In this context, there is an urgent need to discover and develop new antifungal agents as therapeutic alternatives to control these infections [3,7].

The development and obtainment of new drugs is a slow and complex process. The synthesis or derivation of new molecules is the first stage in the discovery of new drug candidates. Thiophenic derivatives are compounds with an aromatic heterocyclic group that is structurally important in several new pharmaceutical and chemical compounds [8]. Specifically, compounds that are derived from 2-aminothiophene display several biological activities that have been already described in the literature, such as antiproliferative activity against human cervical and pancreatic cells [9] as well as antileishmanial [10], anti-inflammatory [11], antibacterial, and antifungal [12,13] activities.

**Figure 1:**
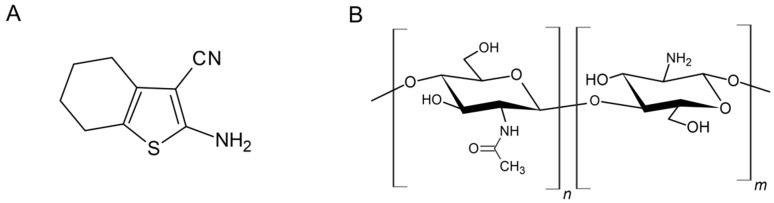
Structural representation of (**A**) 2-amino-4,5,6,7-tetrahydrobenzo[*b*]thiophene-3-carbonitrile (6CN); (**B**) chitosan.

The compound 2-amino-4,5,6,7-tetrahydrobenzo[*b*]thiophene-3-carbonitrile (6CN) (Figure 1A) is a 2-aminothiophene derivative with great pharmacological potential. However, its low solubility in water [14] can decrease its bioavailability. To address this drawback, pharmaceutical technology can be employed to develop novel drug delivery systems that are capable of optimizing the biological activities of poorly soluble compounds, as it has been reported for other bioactive compounds [15,16,17,18].

Polymer matrices that are based on biopolymers have become popular due to their chemical resistance, complete biodegradability, biocompatibility, low toxicity, excellent film-forming properties, and antimicrobial activity. Such biopolymers are widely used in topical preparations as thickening agents, a part of controlled drug delivery systems, wound dressings, and tissue engineering scaffolds [19,20,21].

Chitosan is an acid medium soluble polysaccharide that is obtained from chitin *N*-deacetylation. Its structure consists of *N*-acetyl-d-glucosamine and d-glucosamine units that are arranged depending on the degree of acetylation [22]. Polymeric chitosan films have sparked interest as novel drug delivery and controlled release systems, particularly for topical use. This interest is due to their biodegradability, ease of application, low toxicity, and control of the drug concentration in skin formulations [23,24,25,26].

Considering not only the great chemical versatility and promising biological applications that are associated with the pharmacological potential of 2-aminothiophene derivatives but also their limitations, the present work aims at the development and investigation of the antifungal potential of 6CN-chitosan films, consisting of the incorporation of the active 6CN in a chitosan matrix, as well as evaluating the physicochemical properties of the films.

Unlike some studies that have already reported the use of conventional drugs in polymer matrices [27], our proposal involves the incorporation of a drug prototype into the chitosan matrix, thus contributing to the trajectory of 6CN investigation and transformation into a drug. Similar studies were performed to evaluate the pharmacological potential of other active compounds in polymer matrices for other applications [18,28].

## 2. Results and Discussion

### 2.1. Physicochemical Characterization

#### 2.1.1. Fourier-Transform Infrared Spectroscopy (FTIR)

**Figure 2:**
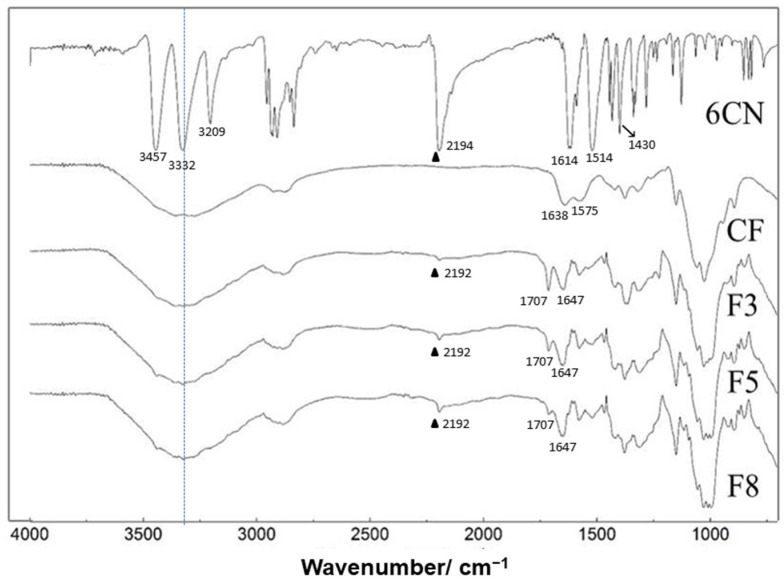
FTIR spectra of 2-amino-4,5,6,7-tetrahydrobenzo[*b*]thiophene-3-carbonitrile (6CN), blank chitosan film (CF), and 6CN-chitosan films with 6CN concentrations of 0.48 mg·mL^−1^ (F3), 0.80 mg·mL^−1^ (F5), and 1.28 mg·mL^−1^ (F8). (▲) C≡N stretching.

Figure 2 shows the FTIR spectra of 2-amino-4,5,6,7-tetrahydrobenzo[*b*]thiophene-3-carbonitrile (6CN), blank chitosan film (CF), and 6CN-chitosan films with 6CN concentrations of 0.48 mg·mL^−1^ (F3), 0.80 mg·mL^−1^ (F5), and 1.28 mg·mL^−1^ (F8).

The spectrum of CF revealed the absorption bands between 3500 and 3300 cm^−1^ that were attributable to O–H stretching, which overlap the N-H stretching in the same region, as well as asymmetric and symmetrical C-H stretching at 2990 and 2850 cm^−1^ [29]. It is also possible to observe a band at 1638 cm^−1^ corresponding to the acetylated amino group of chitin, which indicates partial deacetylation [30]. The absorption band that was observed at 1575 cm^−1^ can be ascribed to the angular deformation of N-H (primary amino group) and that at 1384 cm^−1^ to the alcoholic group C–O stretching [23], while the broad absorption at 1032 cm^−1^ to the R-CH_2_-OH stretching. Finally, the absorption bands in the 1260–800 cm^−1^ range were due to the glycosidic ring, and, in particular, that at 1156 cm^−1^ to the glycosidic bond [31].

The bands in the 3500–3100 cm^−1^ region of the 6CN spectrum were characteristic of the primary amine N-H bond, while the band at 2194 cm^−1^ corresponded to the stretching of the C≡N triple bond. This spectrum also shows bands at 1614 and 1514 cm^−1^ that refer to the stretching of the C=C double bond of the thiophene ring as well as a band at 1430 cm^−1^ that corresponded to the angular bending of the C-H bond of the cyclohexene ring. These bands were all in agreement with the literature [14].

The spectra of 6CN-chitosan films are similar to the one of CF, apart from the additional presence of vibrational modes at 2192 and 1514 cm^−1^, corresponding to the stretching of C≡N and C=C bonds, respectively. Additionally, the band at 1638 cm^−1^ corresponding to chitosan was shifted to 1647 cm^−1^, and a new band appeared at 1707 cm^−1^, possibly due to residual acetic acid whose intensity was reduced in the order of the film F3 to F8, as the active compound concentration increased in the formulation. The absorption band at 1614 cm^−1^, corresponding to the stretching of the C=C double bond of the thiophene ring, was also shifted. Finally, it is possible to observe a reduction in the intensity of the characteristic 6CN bands compared to the spectrum of the isolated compound.

**Figure 3:**
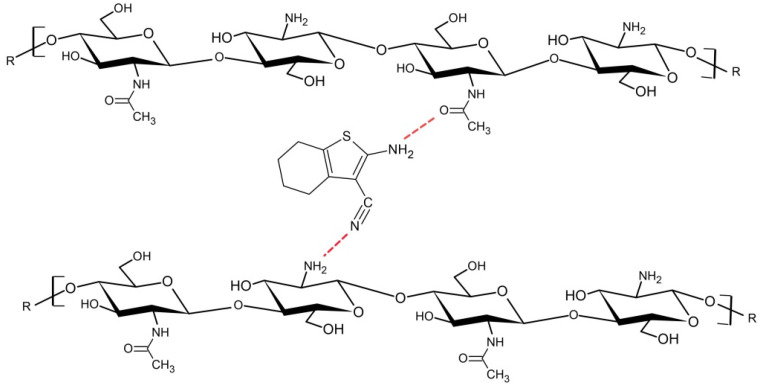
Representation of the possible interactions of 6CN with the polymeric matrix.

According to the spectral changes that were found, 6CN interaction with the polymer matrix can be suggested. In particular, the displacement of the stretching of 6CN C≡N bond from 2194 to 2192 cm^−1^ and that of the stretching of C=O of chitosan acetyl group from 1638 to 1647 cm^−1^, as well as the presence of wide bands in the NH stretching region, suggest the occurrence of possible (1) hydrogen interactions between the chitosan amino groups (-NH_2_) and the 6CN nitrile group, and (2) interactions between the polymer carbonyl and the 6CN amino group, as shown in Figure 3.

#### 2.1.2. Differential Scanning Calorimetry (DSC)

**Figure 4:**
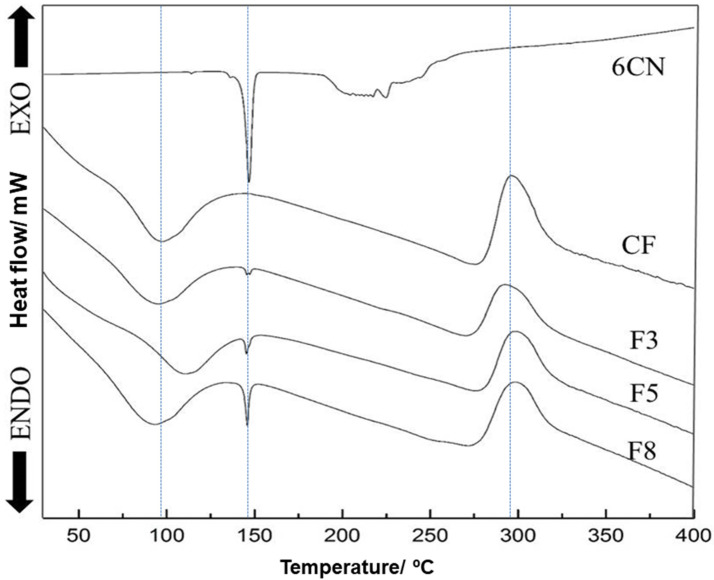
Differential scanning calorimetric curves of 2-amino-4,5,6,7-tetrahydrobenzo[*b*]thiophene-3-carbonitrile (6CN), blank chitosan film (CF), and 6CN-chitosan films with 6CN concentrations of 0.48 mg·mL^−1^ (F3), 0.80 mg·mL^−1^ (F5), and 1.28 mg·mL^−1^ (F8).

The differential scanning calorimetry (DSC) curve of 6CN (Figure 4) reveals two clear endothermic events. The first one that occurred at temperatures between 145 and 151 °C (T_onset_ 145 °C, T_endset_ 151 °C, T_peak_ 148 °C) with a defined melting point, is characteristic of crystalline compounds. On the other hand, the second event that occurred between 188 and 253 °C (T_onset_ 188 °C, T_endset_ 253 °C, T_peak_ 217 °C) can be ascribed to 6CN degradation [14].

The chitosan film exhibited an endothermic event at around 100 °C corresponding to the release of water that was absorbed in the film matrix [32,33] and an exothermic event at approximately 290 °C that was related to the degradation of chitosan [27,28]. In the current study, the glass transition temperature (T_g_) of the chitosan film was not identified, corroborating other works [32,33]. Despite the T_g_ of polymers being analyzed in the DSC and chitosan being widely investigated in the literature, its T_g_ results are controversial. In fact, some specific characteristics of the chitosan molecule, such as the degree of deacetylation and the molecular weight, can change the glass transition temperature [32,34].

The DSC curves of 6CN-chitosan films that are illustrated in the Figure 4 show a first event that is close to 100 °C that is probably due to polymer dehydration. Some changes were found for this event in relation to T_peak_, in that the F3 film had a T_peak_ of 96 °C, while the F5 one had a T_peak_ shift to 110 °C that was possibly due to a greater interaction between the chitosan chains as a result of chitosan-6CN-chitosan interactions. On the other hand, it is suggested that in the F8 film there was a greater distance between the chitosan chains compared to the F5 film due to the interactions between the 6CN molecules themselves, thus allowing a greater heat flux between the polymer chains, resulting in the T_peak_ of 92 °C. A similar effect was reported for chitosan films with added minerals or vitamins, in which the endothermic dehydration peak was shifted to lower temperatures with increasing concentration [33].

Additionally, a second endothermic event was verified for films between 144 and 150 °C (T_onset_ 144 °C, T_endset_ 150 °C, T_peak_ 148 °C) that corresponds to the 6CN melting peak and is the more evident the higher its concentration in the films. A third event of the exothermic type that occurred in the temperature range from 280 to 320 °C can be attributed to polymer degradation, while thermal degradation of 6CN in the films was not clearly evident in the thermograms.

The crystallinity of the films was reduced compared to that of the isolated 6CN, considering the amount of 6CN that was present in the new 6CN-chitosan films. Furthermore, the incorporation of the active compound into the polymeric matrix can mask the melting peak corresponding to 6CN [13].

#### 2.1.3. Thermogravimetric Analysis (TGA)

**Figure 5:**
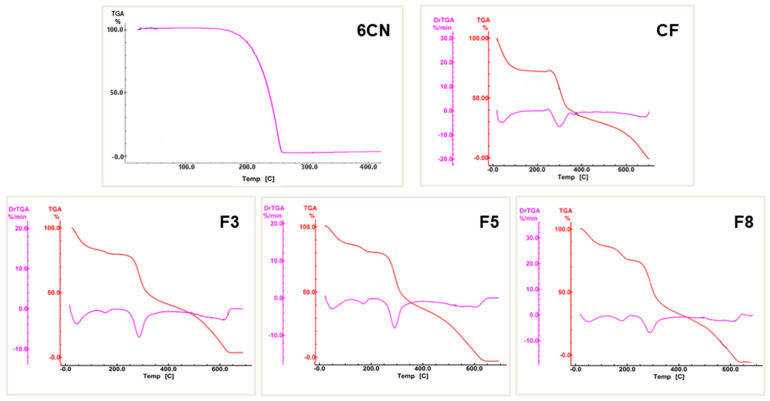
Thermogravimetric curves of 2-amino-4,5,6,7-tetrahydrobenzo[*b*]thiophene-3-carbonitrile (6CN), blank chitosan film (CF), and 6CN-chitosan films with 6CN concentrations of 0.48 mg·mL^−1^ (F3), 0.80 mg·mL^−1^ (F5), and 1.28 mg·mL^−1^ (F8).

The thermogravimetric curve of 6CN (Figure 5) shows only one defined event at temperatures between 146 and 250 °C, with a 98.27% mass loss (Δm) due to its degradation.

In the TGA curve of the CF film, three stages of mass loss can be observed, the first was between 35 and 120 °C with 26.23% mass loss (Δm), referring to the elimination of the absorbed water from the polymer. A second stage that was between 250 and 355 °C (Δm = 32.10%) is characteristic of the thermal degradation of chitosan, i.e., breaking of the polymer chain, depolymerization, and decomposition of its acetylated and deacetylated units [35]. The third stage that was between 355 and 700 °C (Δm = 38.00%), refers to the degradation of the products that were generated in the previous event.

In turn, the 6CN-chitosan films exhibited four stages of mass loss, as reported in Table 1. The first stage that occurred in the range 35–120 °C, corresponded to dehydration and led to mass losses of 15.35% (F3), 12.10% (F5), and 11.10% (F8). The second stage, which took place in the range between 146 and 240 °C, corresponded to 6CN degradation, with mass losses of 4.25% (F3), 6.21% (F5), and 10.77% (F8), and indicated the active compound incorporation into the polymer matrix.

However, the greatest mass losses that occurred in the ranges 240–350 °C (third stage) and 350–640 °C (fourth stage) were attributed to the degradation of chitosan and its by-products. Particularly, the F3, F5, and F8 films showed mass losses of 32.43%, 36.87%, and 35.62%, respectively, referring to the third stage, and 43.62%, 43.90%, and 41.25%, respectively, referring to the fourth one.

Analyzing the TGA curves of the 6CN-chitosan films, it was verified that the presence of 6CN in the formulation of the films resulted in a decrease in the presence of absorbed water inside the polymer, corroborating reports in the literature [36]. The F8 film with the highest concentration of the active compound exhibited a lower percentage of water loss (Δm = 11.10%) compared to the CF film (Δm = 26.23%). Additionally, a mass loss stage was found in the 6CN degradation range, which demonstrates its incorporation into the obtained films.

#### 2.1.4. X-ray Diffraction (XRD)

**Figure 6:**
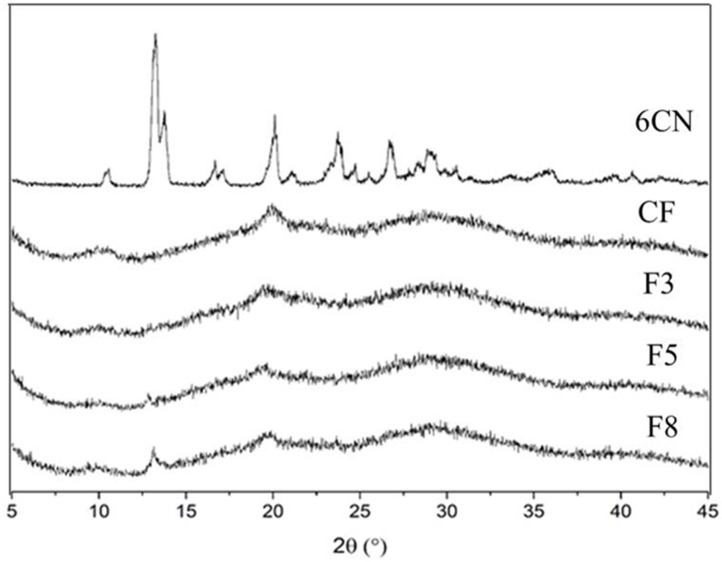
X-ray diffraction patterns of 2-amino-4,5,6,7-tetrahydrobenzo[*b*]thiophene-3-carbonitrile (6CN), blank chitosan film (CF), and 6CN-chitosan films with 6CN concentrations of 0.48 mg·mL^−1^ (F3), 0.80 mg·mL^−1^ (F5), and 1.28 mg·mL^−1^ (F8).

6CN X-ray diffraction (XRD) pattern (Figure 6) points out several crystalline reflections. This is characteristic of a crystalline compound and is supported by major reflections around 13° and others of less intensity at approximately 20°, 23°, 26° and 29°. This result corroborates the well-defined melting point that was identified by DSC.

CF exhibited low crystalline reflections with some weak diffraction peaks centered at 20°, which can be attributed to the usually amorphous state of CF [29]. 6CN-chitosan films presented diffraction halos that were characteristic of amorphous structures, as well as crystalline reflections that started at 10° and 20°, that were characteristic of the chitosan diffractogram. F5 and F8 films were partially amorphous which is in agreement with the results of DSC, confirming the presence of 6CN and due to their higher 6CN contents compared to F3.

#### 2.1.5. Scanning Electron Microscopy (SEM)

**Figure 7:**
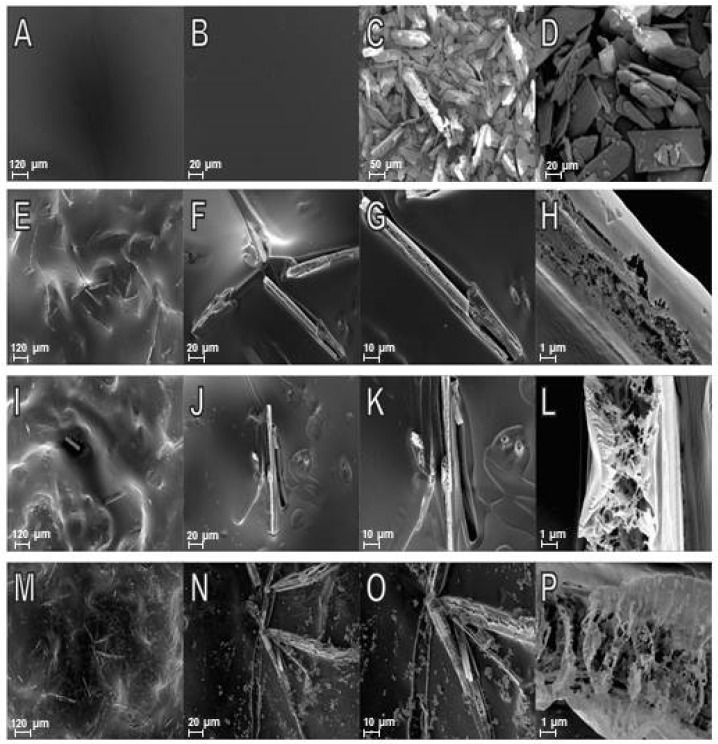
SEM micrographs of blank chitosan film at (**A**) 100× and (**B**) 500×; of 2-amino-4,5,6,7-tetrahydrobenzo[*b*]thiophene-3-carbonitrile (6CN) at (**C**) 150× and (**D**) 500×; of 6CN-chitosan films with 6CN concentration of 0.48 mg·mL^−1^ (F3) at (**E**) 100×, (**F**) 500×, (**G**) 1000×, and (**H**) 10,000×; of 6CN-chitosan films with 6CN concentration of 0.80 mg·mL^−1^ (F5) at (**I**) 100×, (**J**) 500×, (**K**) 1000×, and (**L**) 10,000×; of 6CN-chitosan films with 6CN concentration of 1.28 mg·mL^−1^ (F8) at (**M**) 100×, (**N**) 500×, (**O**) 1000×, and (**P**) 10,000×.

Scanning electron microscopy (SEM) analysis revealed that CF was uniform with no pores or cracks (Figure 7A,B), corroborating the smooth, continuous and compact structure that was already reported for chitosan films [20]. The 6CN micrograph shows particles of defined crystalline aspect with multilayer structured crystals of different shapes and sizes (Figure 7C,D); this is in agreement with the several diffraction peaks that were revealed by the XRD analysis. SEM micrographs of the 6CN-chitosan films showed that an increase in 6CN concentration caused growth, increased aggregation, and increased the number of free-form major crystals on the polymeric matrix surface (Figure 7E–P). According to DSC and XRD results, the melting point appears in a more evident way at higher concentrations.

### 2.2. Film Thickness

The thickness of the 6CN-chitosan films exhibited similar mean values (around 57 ± 4 µm), resulting in thin films. According to the literature, the film thickness is an important parameter that can influence the performance of films in their applications, including in the pharmaceutical area such as drug delivery systems [18].

### 2.3. Drug Content in 6CN-Chitosan Films

Based on the mass of 6CN that was found by spectrophotometric method on film strips (size of 15 mm × 15 mm) and reported in the Table 2, it was possible to determine the drug content in the films. The analysis of the 6CN content in the films revealed that the F8 formulation had higher drug content (16.91% *w*/*w*) than the F3 (5.30% *w*/*w*) and F5 (10.52% *w*/*w*) ones (Table 2).

These results agree with the SEM images and with what was expected from the increasing concentration of 6CN that was used to prepare the films from F3 to F8.

### 2.4. Antifungal Activity of 6CN-Chitosan Films

**Figure 8:**
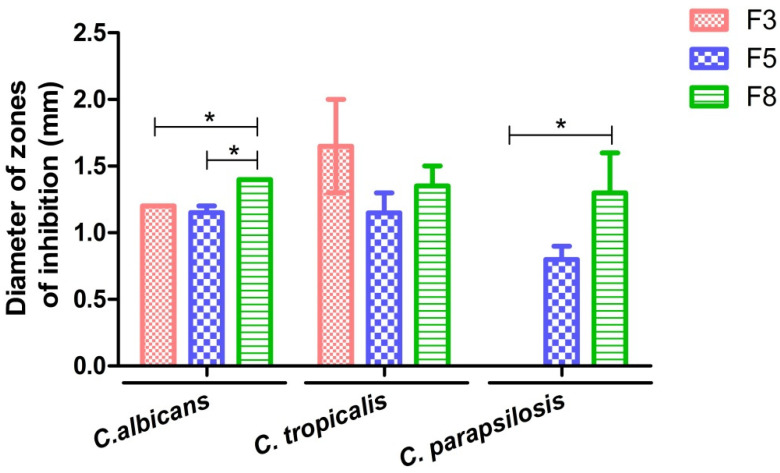
Diameter of the inhibition zones that were caused by 6CN-chitosan films (F3, F5, and F8) against *Candida albicans*, *Candida tropicalis*, and *Candida parapsilosis*. Values were expressed as the mean ± standard error of mean. (*n* = 3, * *p* < 0.05).

Figure 8 shows the diameters of the inhibition zones that were caused by 6CN-chitosan films on the growth of selected the target microorganisms, namely *Candida parapsilosis*, *Candida albicans*, and *Candida tropicalis*. All the films produced inhibition zones, except the F3 one against *C. parapsilosis*, indicating a drug concentration-dependent inhibitory effect on microbial growth. The greatest susceptibility of all three species was, in fact, observed with the F8 film that was prepared with the highest 6CN concentration.

Although chitosan has been reported to have antimicrobial activity against a variety of Gram-positive and Gram-negative bacteria and fungi [37], it did not show any inhibitory effects on the yeasts that were tested in this study, confirming previous findings that were reported on the effect of this polymer against *Pseudomonas aeruginosa* [22]. Several factors can influence the activity of chitosan against fungi and bacteria, such as the size of the polymeric chains and variations in pH and temperature, which may explain these variations in the antimicrobial activity of the polymeric matrix [38].

Based on these results we can then infer that the 6CN that is present in the chitosan matrix was responsible for the fungal inhibition that was caused by the films. Consistently, with the antifungal activities of thiocompounds such as thiosemicarbazones [39,40] and thiophene-thiosemicarbazone derivatives, the latter showed promising antifungal activity, mainly against *C. neoformans* [5].

## 3. Materials and Methods

### 3.1. Materials

The compound 2-Amino-4,5,6,7-tetrahydrobenzo[*b*]thiophene-3-carbonitrile (6CN) (C_9_H_10_N_2_S, MW = 178.25 g·mol^−1^, ≥95% purity) was synthesized and provided by the Molecule Synthesis and Vectoring Laboratory of the State University of Paraíba. Chitosan (Product Number: 448877) (deacetylation degree above 75–85% and molecular weight of 190,000–310,000 Da) was obtained from Sigma-Aldrich (St. Louis, MO, USA). Ethanol was purchased from Vetec (Duque de Caxias, Brazil) and the other reagents and solvents were of analytical grade.

### 3.2. Development of Chitosan-Based Films

The blank chitosan and 6CN-chitosan films were prepared using the solvent casting method as described in the literature with some adaptations [18]. Polymer solutions (1% *w*/*v*) were prepared by the dissolution of chitosan in diluted acetic acid (1% *v*/*v*) under magnetic stirring for 24 h at room temperature (25 ± 0.5 °C). To obtain a blank chitosan film (CF), 15 mL of the polymeric solution was poured into 47-mm diameter Petri dishes (area = 17.3 cm^2^) followed by drying for 24 h in an incubator shaker at 50 rpm and 50 °C.

To obtain 6CN-chitosan films, 6CN solutions at three concentrations (3, 5, and 8 mg·mL^−1^) were prepared in ethanol. Subsequently, 12.6 mL of the chitosan solution were added to 2.4 mL of the 6CN solution (3, 5 and 8 mg·mL^−1^) to obtain the 6CN-chitosan films called F3, F5, and F8, respectively, with concentrations of 0.48, 0.80, and 1.28 mg·mL^−1^ of 6CN. The obtained solution was homogenized under magnetic stirring at 100 rpm, poured into 47-mm diameter Petri dishes and dried in the same way as CF. After drying, both the chitosan and 6CN-chitosan films were immersed in sodium hydroxide solution (1 M) for 30 min to neutralize any acid residue, followed by three washings with distilled water to ensure neutrality (pH = 7.0). The films exhibited a translucent aspect.

### 3.3. Physicochemical Analyses

#### 3.3.1. Fourier-Transform Infrared Spectroscopy (FTIR)

FTIR analyses were performed on an IR Prestige-21 spectrophotometer (Shimadzu, Tokyo, Japan). 6CN (in solid state) and the films were placed on steel plates and analyzed directly by attenuated total reflectance (ATR). The analyses were carried out within the wavenumber range of 4000–700 cm^−1^, with 15 scans and spectral resolution of 4 cm^−1^.

#### 3.3.2. Differential Scanning Calorimetry (DSC)

The DSC analyses were performed using a DSC-60A calorimeter (Shimadzu, Tokyo, Japan), employing aluminum crucibles with approximately 3 mg of sample, under a nitrogen atmosphere (50 mL·min^−1^), and heating rate of 10 °C·min^−1^ in the temperature range of 25–400 °C. The DSC instrument was calibrated with Indium standard.

#### 3.3.3. Thermogravimetric Analysis (TGA)

TGA curves were obtained with a DTG 60AH thermobalance (Shimadzu, Tokyo, Japan). Alumina crucibles were used with approximately 3 mg of sample under nitrogen atmosphere (50 mL·min^−1^) and a heating rate of 10 °C·min^−1^. The temperature range was 20–700 °C. The thermobalance was calibrated with CaC_2_O_4_·H_2_O standard.

#### 3.3.4. X-ray Diffraction (XRD)

XRD analyses were carried out on a D2 Phaser diffractometer (Bruker Corporation, Billerica, MA, USA), with CuKα radiation (λ = 1.54 Å) at a voltage of 30 kV and a current of 10 mA using a Lynxeye detector (Bruker Corporation, Billerica, MA, USA). The analyses were performed with a 2° angle in the region between 5 and 40° with step size of 0.05° and scan speed of 5°·min^−1^.

#### 3.3.5. Scanning Electronic Microscopy (SEM)

Double-sided carbon tape was used to fix the samples in the sample holder, and metallization with gold that was performed for one minute and with 30 mA on the SCD 005 Sputter coater (BAL-TEC, Leica Biosystems, Wetzlar, Germany). Morphological analyses were performed on a scanning electron microscope with field emission gun (SEM-FEG), model Auriga (Carl Zeiss, Oberkochen, Germany), at magnifications of 100×, 500×, 1000×, and 10,000×. The SEM images were obtained at an accelerating potential of 5 kV under reduced pressure.

### 3.4. Film Thickness

The film thickness was measured with the aid of a scanning electronic microscope (SEM) by taking the image of the cross section. The measurements were made at nine random points and reported as a mean.

### 3.5. Drug Content of Films

The amount of 6CN that was loaded in each film strip (size of 15 mm × 15 mm) was determined spectrophotometrically. For this purpose, the film samples were weighed and dissolved in 5 mL of a 1% (*v*/*v*) acetic acid solution under magnetic stirring for 1 h, and subsequently diluted in phosphate buffer pH 7.4/ethanol (50:50, *v*/*v*). Then, the absorbance was measured with a spectrophotometer at a wavelength of 243 nm. All the analyses were conducted in triplicate. The percentage of the drug content of the film was calculated by the equation:

Drug content % (*w*/*w*) = (mass of 6CN/film mass) × 100 (1)

The spectrophotometric method was validated for selectivity, linearity, detection and quantification limit, precision, and accuracy, according to the International Conference of Harmonization (ICH) [41]. The drug content was determined using a calibration curve showing linearity over the 6–30 μg·mL^−1^ range, (y = 0.0268x + 0.0864) (R² = 0.9995). The limits of detection (LOD) and quantitation (LOQ) were 0.05 μg·mL^−1^ and 0.17 μg·mL^−1^, respectively.

### 3.6. Antifungal Activity

The antifungal activity of the films was determined using the disc diffusion method, according to CLSI [42]. The strains *Candida parapsilosis* (ATCC 22019), *Candida tropicalis* (URM 4262), and *Candida albicans* (URM 5744) were used as target microorganisms.

The fungi strains were seeded 24 h (overnight) before analysis in Saboraud culture medium. For inoculum preparation, one colony from each culture was taken and transferred to a tube containing 10 mL of 0.85% sterile saline solution. The optical density was employed to adjust the turbidity to 0.5 standard of the McFarland scale (corresponding to 1 to 5 × 10^6^ CFU·mL^−1^) on an UV-Vis spectrophotometer (Shimadzu, Tokyo, Japan) at a wavelength of 530 nm.

After the inoculum standardization with approximately 1 to 5 × 10^6^ CFU·mL^−1^, the fungi were seeded in Petri dishes containing Muller Hinton agar (Kasvi, São José dos Pinhais, Brazil) using a sterile swab. The 6CN-chitosan films (F3, F5, and F8) and blank chitosan film (CF) with a thickness of 57 ± 4 µm were used as the discs, being cut into a circular shape with a diameter equal to 6 mm, a size that was equivalent to the antibiogram discs. Subsequently, the 6CN-chitosan films and blank chitosan film (negative control) were added to the Petri dishes. All the tests were performed in triplicate and incubated at 35 °C for 24 h. The diameter of the inhibition zone of samples was measured using a caliper.

### 3.7. Statistical Analysis

The antifungal activity data were expressed as the mean ± standard error of the mean. Statistical analysis was done by one-way analysis of variance (ANOVA) followed by Bonferroni’s multiple comparison test, using the GraphPad Prism software version 5.0 (La Jolla, CA, USA). Values of *p* < 0.05 were considered statistically significant.

## 4. Conclusions

The chitosan films that were loaded with 2-amino-4,5,6,7-tetrahydrobenzo[*b*]thiophene-3-carbonitrile (6CN) were successfully developed by the methodology that was proposed in this study. The studies that were carried out by FTIR, XRD, DSC, and TGA indicated that 6CN-chitosan films can lead to modifications in the physicochemical characteristics of 6CN. The crystallinity of the films was reduced compared to the crystallinity of the isolated 6CN, considering the practically amorphous character of chitosan and the amount of 6CN present in the new films. The morphological images suggested that the 6CN particles were relatively well dispersed in the chitosan matrix. Furthermore, the 6CN-chitosan films showed an antifungal effect. The films containing 6CN at concentrations of 0.80 mg·mL^−1^ (F5) and 1.28 mg·mL^−1^ (F8) exhibited antifungal activity against *Candida albicans, Candida tropicalis*, and *Candida parapsilosis*, while the one with the lowest drug concentration (0.48 mg·mL^−1^, F3) did so only against the first two species, thus demonstrating a drug concentration-dependent pattern. From the results of this study, one can infer that the incorporation of 6CN into the films proved to be a promising strategy for antifungal candidates.

## Figures and Tables

**Table 1:** Data that were obtained by thermogravimetric analysis (TGA).

Sample	Loss Stage	Temperature (°C)	Mass Loss (%)	Process
6CN	First	146–250	98.27	Degradation 6CN
CF	First	35–120	26.23	Dehydration
	Second	250–355	32.10	Degradation/depolymerization
	Third	355–700	38.00	Degradation
F3	First	35–120	15.35	Dehydration
	Second	146–240	4.25	6CN degradation
	Third	240–350	32.43	Degradation/depolymerization
	Fourth	350–640	43.62	Degradation
F5	First	35–120	12.10	Dehydration
	Second	146–240	6.21	6CN degradation
	Third	240–350	36.87	Degradation/depolymerization
	Fourth	350–640	43.90	Degradation
F8	First	35–120	11.10	Dehydration
	Second	146–240	10.77	6CN degradation
	Third	240–350	35.62	Degradation/depolymerization
	Fourth	350–640	41.25	Degradation

**Table 2:** Characteristics of the 6CN-chitosan films.

Parameter	F3	F5	F8
Film mass (size 15 mm × 15 mm) (mg)	19.07 ± 1.21	19.50 ± 1.90	19.70 ± 1.87
* mass of 6CN (mg)	1.01 ± 0.07	2.03 ± 0.09	3.35 ± 0.60
6CN content (% *w*/*w*)	5.30 ± 0.36	10.52 ± 1.40	16.91 ± 1.74

Values presented as the mean ± SD, *n* = 3. * mass of 6CN contained in film samples with 15 mm × 15 mm size.

## Data Availability

The data presented in this study are available on request from the corresponding author. The data are not publicly available due to privacy.
